# Spontaneous Retroperitoneal Hemorrhage in a Patient with Acquired Cystic Kidney Disease

**DOI:** 10.15586/jkcvhl.2020.123

**Published:** 2020-04-16

**Authors:** Ahmed Kotb, Asmaa Ismail, Hazem Elmansy, Owen Prowse, Walid Shahrour

**Affiliations:** Northern Ontario School of Medicine, Thunder Bay Regional Health Sciences Centre

**Keywords:** hemodialysis, lumbotomy, retroperitoneal hemorrhage

## Abstract

Spontaneous retroperitoneal hemorrhage (SRH) is a rare emergency. It is usually encountered in patients on hemodialysis and is associated with high rate of morbidity and mortality. This is a case from the emergency department. The patient had unstable vitals with SRH following dialysis. Immediate exploration and nephrectomy using transverse lateral lumbotomy incision were done. Patients on hemodialysis are at a risk of SRH and frequent surveillance is recommended. Acquired cystic kidney disease (ACKD) can develop in hemodialysis patients and put them at risk for bleeding. Transverse lateral lumbotomy may be a safe option for direct access to the kidney in emergency kidney surgery

## Introduction

Spontaneous retroperitoneal hemorrhage (SRH), following hemodialysis, is rare and can be associated with high risk of morbidity and mortality. Most hemodialysis patients receive anticoagulants that put them at risk of bleeding diathesis. Diagnostic imaging of the hemodialysis patient is best done by using computed tomography (CT) scan (1). Acquired cystic kidney disease (ACKD) can develop in patients on hemodialysis. Two significant problems related to this pathology are the association with SRH and the ACKD-associated renal cell carcinoma (RCC). A recent study found that the presence of ACKD in dialysis patients has 35 times higher risk for the development of RCC than any other group with renal failure (2).

## Case Report

An 85-year-old female patient presented to the emergency department 3 h after receiving hemodialysis, with severe left loin pain and dizziness. Her blood pressure was 70/40 and pulse was 110 not responding to bolus infusions of normal saline. Serum hemoglobin was 60 g/L. Examination revealed a left hypochondrial tender mass, with clinical evidence of hemorrhagic shock. An emergent CT of the abdomen and pelvis was done and showed extensive left retroperitoneal hemorrhage and heterogeneous left kidney. The patient was on warfarin and had end-stage renal disease (ESRD) secondary to diabetes mellitus, atrial fibrillation, congestive heart failure, diastolic dysfunction, coronary artery bypass grafting, and hypertension.

She was positioned with left flank elevated 45° and left retroperitoneal subcostal transverse lateral lumbotomy was done. The retroperitoneal cavity was full of blood clots that were removed; this was rapidly followed by kidney dissection using blunt dissection and Ligasure vascular control. The renal pedicle was soon accessible and Hem-o-lok clips were used for pedicle control. The kidney was safely removed and the whole procedure was completed in 35 min. The patient vitals were stabilized and she was transferred to ICU for 24 h observation.

Pathology showed extensive sclerosed glomeruli, marked interstitial inflammation, severe arteriosclerosis, perirenal and renal parenchymal hemorrhage, severe renal artery atherosclerosis, and ACKD. Figurers 1 and 2 show the enlarged left kidney with massive retroperitoneal hemorrhage on CT scan.

**Figure 1:**
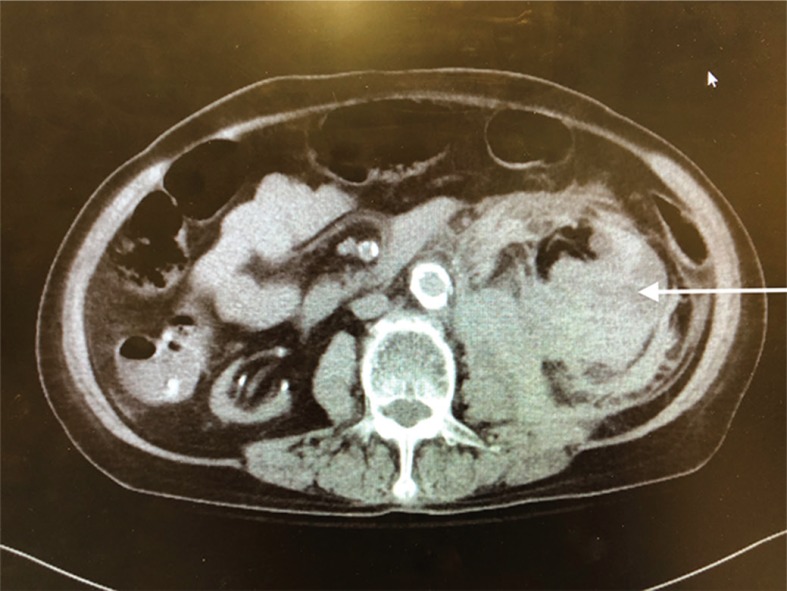
Axial CT image of left retroperitoneal hemorrhage.The arrow refers to the large retroperitoneal hematoma.

**Figure 2:**
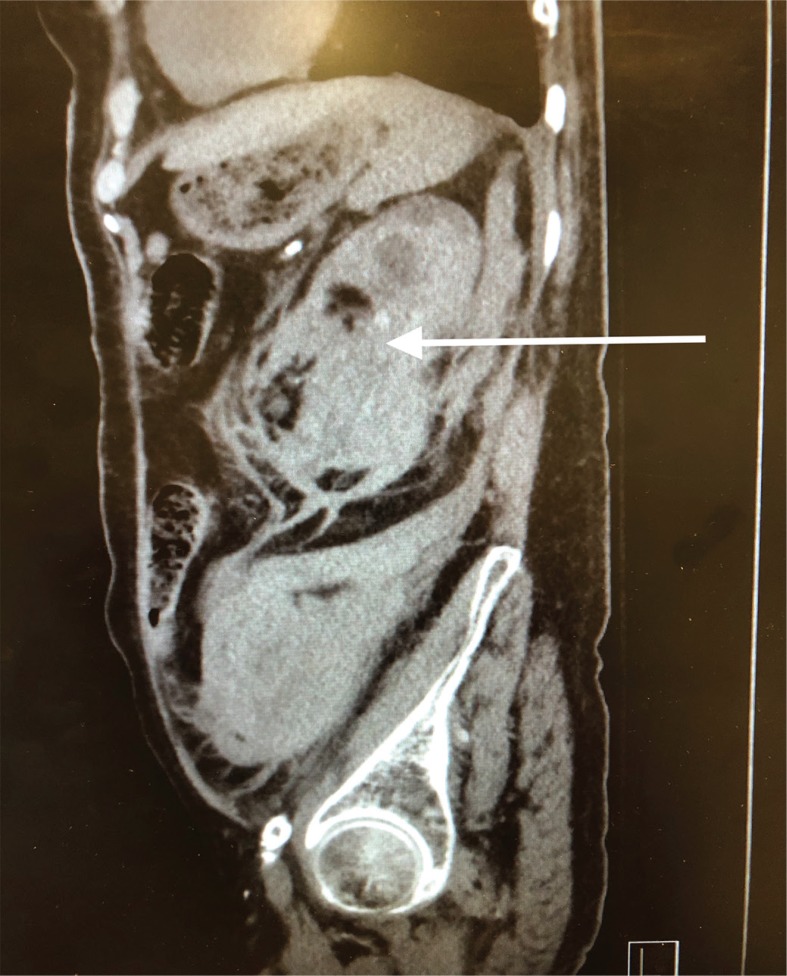
Sagital CT image of left retroperitoneal hemorrhage. The arrow shows the extensive left retroperitoneal hematoma with rupture of the left kidney.

## Discussion

SRH is a rare emergent condition occasionally presenting to the emergency department. When these patients present to the emergency department, they are usually dialysis patients. A meta-analysis looking for all publications over 14 years could find 165 cases, with RCC and angiomyolipoma representing the most common causes (1). Kim et al. did a retrospective study of patients presenting with SRH in their department between 2011 and 2018 and could only identify six patients presented while on dialysis for ESRD (3).

Murphy et al. described a case of 55-year-old patient presenting to the emergency department, following hemodialysis, with left loin pain and hemorrhagic shock. Exploratory laparotomy was done and left nephrectomy was completed. The pathological analysis showed ACKD (4). Biyik et al. reported the case of a 44-year-old male patient presenting with SRH, while on peritoneal dialysis, necessitating nephrectomy, and they confirmed the necessity for surveillance of these patients for ACKD that put them at higher risk of renal bleeding (5). Xie et al. reported three cases presented with SRH post-hemodialysis and referred to ACKD as a risk factor of renal bleeding (6). Fan et al. reported a 67-year-old woman presenting with SRH and managed conservatively and raised awareness of the possibility of diabetic nephropathy being the main risk of this bleeding in dialysis patients receiving anticoagulants (7).

At present there are no proper guidelines to approach patients with life-threatening SRH. Only conservative management can rarely be successful for these patients, and intervention is usually needed to avoid high mortality risk. Angioembolization is emerging as a less invasive way of management. Wang et al. did a retrospective study for patients diagnosed with SRH over a 10-year period. They identified 16 patients, of which seven managed conservatively, seven had successful embolization, and two died. The mean age of their patients was 51.5 years (4). Murphy et al. published a case report for a dialysis patient presenting with SRH and, similar to us, they chose surgical approach to effectively manage their patient (8). Interestingly, Li et al. published a randomized clinical trial comparing surgical packing versus embolization for patients presenting with massive hemorrhage related to pelvic fracture. They found that packing was significantly associated with shorter time to manage the patient than embolization. Two patients in angioembolization group died from persistent hemorrhage, while no single patient in packing group died of bleeding (9).

Our patient was a frail older female patient with multiple comorbidities. She had severe massive hemorrhage, not responding to conservative management. Surgery was the fastest effective way for bleeding control. We recently published our approach for nephrectomy (10), which we used for this case and allowed direct access to the kidney and left retroperitoneal space, allowing fast safe nephrectomy and hematoma evacuation while avoiding the risk of pleural injury as well as higher morbidities associated with transperitoneal exploration.

## Conclusion

Patients on hemodialysis are at a risk of SRH and frequent surveillance for ACKD is recommended. Subcostal transverse lumbotomy may be a safe option for direct access to the kidney and retroperitoneal space in emergency situations requires nephrectomy.
